# Editorial: Identifying Neuroimaging-Based Markers for Distinguishing Brain Disorders

**DOI:** 10.3389/fnins.2020.00327

**Published:** 2020-04-08

**Authors:** Yuhui Du, Jing Sui, Dongdong Lin

**Affiliations:** ^1^School of Computer and Information Technology, Shanxi University, Taiyuan, China; ^2^Tri-Institutional Center for Translational Research in Neuroimaging and Data Science (TReNDS), Georgia State University, Atlanta, GA, United States; ^3^Chinese Academy of Sciences (CAS) Centre for Excellence in Brain Science and Intelligence Technology, University of Chinese Academy of Sciences, Beijing, China; ^4^Institute of Automation, Chinese Academy of Sciences, University of Chinese Academy of Sciences, Beijing, China

**Keywords:** magnetic resonance imaging (MRI), electroencephalography (EEG), positron emission tomography (PET), diffusion tensor image (DTI), brain connectivity/network, biomarkers

The current diagnosis of brain disorders heavily relies on clinical presentation. Neuroimaging is gaining more importance with the potential to provide useful markers in revealing biological substrates and benefit brain disorder diagnosis. Magnetic resonance imaging (MRI), electroencephalography (EEG), positron emission tomography (PET), diffusion tensor image (DTI), and magnetoencephalography (MEG) have been widely applied to measure the brain structure, decode the brain function, and explore the disease mechanism from different aspects. This Research Topic takes action by publishing 24 papers that proposed new methods for identifying biomarkers from these modalities and utilized neuroimaging measures to differentiate between patients with brain disorders or differentiate patients from healthy controls. Papers in the topic involved different disorders such as schizophrenia (SZ), autism spectrum disorder (ASD), Alzheimer's disease (AD), attention-deficit/hyperactivity disorder (ADHD), and epilepsy.

Resting-state functional MRI (fMRI) has been successful in estimating brain functional networks and connectivity via data-driven methods (Calhoun et al., 2001; Beckmann et al., 2005; Du and Fan, 2013), providing features for the classification between various brain disorders and the prediction of disorder progression (Du et al., 2015, 2018b; Arbabshirani et al., 2017). There has been evidence that brain functional connectivity is time-varying, and clustering (e.g., K-means) and decomposition methods can be used to extract connectivity states from dynamic connectivity patterns (Hutchison et al., 2013; Allen et al., 2014; Calhoun et al., 2014; Preti et al., 2017). Most previous dynamic connectivity studies focused on the dynamics of the connectivity between different brain regions or networks (Damaraju et al., 2014; Yu et al., 2015; Du et al., 2016, 2017, 2018a). A study by Bhinge et al. proposed a novel approach to measure both the voxelwise spatial variability in functional networks and the dynamic functional network connectivity (dFNC). Time-varying spatial networks were estimated by a constrained independent vector analysis. Their method successfully captured distinct information between healthy controls and SZ patients, resulting in relatively high classification accuracy by using dynamic spatial information. Another shortcoming of previous dynamic analyses is that clustering was often performed to all time-varying connectivity matrices without considering their temporal relationship. In the topic, Espinoza et al. incorporated the temporal variation of functional network connectivity into clustering, thus providing more information than regular dFNC method in investigating differences between SZ patients and healthy controls. In another study, Zhao et al. also focused on improving the clustering performance in identifying reliable connectivity states from dynamic connectivity. They used the node centrality of brain regions rather than the original functional connectivity strengths as features, showing that repeatable dynamic features can be found between repeated scans. All these new methods would benefit the biomarker identification from dynamic functional connectivity. On the other hand, potential neuroimaging biomarkers are most meaningful when they can be replicable and used to predict new subjects in clinical practice (Jiang et al., 2018). A previous study (Sun et al., in press) proposed a connectome-based predictive model that can be used to predict depressive rating changes and remission status of major depressive disorder (MDD) patients. In the topic, Zhu et al. identified abnormal brain connections in the lateral habenula and thalamus, and found that they may serve as connectome-based biomarkers to predict the precursor to MDD. Luo et al. investigated the MDD in terms of the functional connectivity between the brainstem regions and other brain regions, providing a new insight for the neurobiology of MDD. Cui et al. proposed a method to integrate local and global properties of brain functional networks for improving the classification performance between early mild cognitive impairment (EMCI) and healthy control groups, based on the minimum spanning tree and graph kernel techniques. A work from Yang et al. obtained high classification accuracy by fusing amplitude of low frequency fluctuation (ALFF) and fractional ALFF features for distinguishing individuals with subjective cognitive decline, patients with amnestic mild cognitive impairment, patients with AD, and healthy controls. Xu, Yang et al. investigated the altered resting-state whole brain functional connectivity in premature ejaculation patients compared to healthy controls via a classification method. Using fMRI, network topological property is one of the most important techniques to elucidate the brain function (Wang et al., 2010; Bullmore and Sporns, 2012). One study by Liu et al. revealed the alterations in diabetes mellitus patients using long-range and short-range functional connectivity degree. Xu, Guo et al. compared the topological properties between diabetes mellitus patients and healthy controls using fMRI connectivity. Wang, Tao et al. studied the topology of frontal parietal attention network in children with ADHD using a graph theory analysis method including the minimum spanning tree technique.

Besides fMRI, other modalities including structural MRI (sMRI), EEG, PET, and MEG can be utilized to provide useful indicators. Xu, Chen et al. reported the first study to show structural and functional brain abnormalities in patients with hemifacial spasm using both fMRI and sMRI. Long et al. found that various brain parcellation schemes may result in different classification performance by using voxel-based morphometry measures summarized in brain regions as features in classifying MCI patients and healthy controls. Yan et al. proposed a new matrix regression method that showed a promise in predicting cognitive data of AD using voxel-based morphometry. Zeng et al. focused on the prediction of medication response in herpes zoster patients by applying a searchlight algorithm and support vector machine on the voxel-based brain morphometry measures. A paper by Ma et al. showed the tissue-specific changes in gray matter and white matter of the mouse model of tauopathy based on the *in vivo* and *ex vivo* conditions, emphasizing the importance of longitudinal analysis. Based on DTI data, Qin et al. applied the graph theory and network-based statistic methods to compare the impairments between obsessive-compulsive disorder and SZ. Wang, Li et al. revealed the abnormality in the hemispheric topological asymmetries in bipolar disorder using DTI-based network analysis. Using both DTI and fMRI network measures, Park et al. reported the changes of individuals with eating disorder and found the brain regions associated with the behaviors. In another study, Hu et al. used the partial least square technique to aid the minimum variance beamforming approach for source imaging with MEG arrays, and verified its effectiveness in simulated data and epilepsy data. Using EEG activity, Simões et al. identified group differences between patients with ASD and healthy controls under the visual stimulation and mental imagery tasks, revealing a possible biomarker of face emotional imagery network of ASD. Shah et al. explored the possible mechanism of depression in human immunodeficiency virus (HIV) by analyzing the longitudinal PET images of an animal model of HIV.

Since different types of neuroimaging techniques reflect the brain's function and structure from different angles, it has been largely acknowledged that through the fusion of complementary information from different modalities, biomarkers of mental illness may be identified more precisely (Sui et al., 2018). Efficient methods that can draw valid conclusions from high dimensional multimodal imaging, cognitive or genetic data are urgently needed (Calhoun and Sui, 2016; Qi et al., 2019). In the topic, Acar et al. applied an advanced coupled matrix and tensor factorizations (CMTF) method to the data of EEG, fMRI, and sMRI collected from patients with schizophrenia and healthy controls to reveal linked biomarkers across different modalities. Compared to joint ICA, they revealed more meaningful and reproducible biomarkers. Besides the neuroimaging studies on brain disorders, increasing work has recognized the role of genetics in the etiology of many complex disorders (e.g., schizophrenia; Lin et al., 2018; Chen et al., 2019). Imaging genetics, a rising field to bridge genetics and neuroimaging, aims to investigate the genetic risk of various imaging endophenotypes in relation to diseases, and identify biomarkers (genetic and imaging) to facilitate the disease diagnosis (Lin et al., 2014). In the topic, Jiang et al. reviewed the current imaging genetics studies on schizophrenia, particularly in revealing the heterogeneity within schizophrenia, and also discussed the potential of imaging genetics in refining disease diagnosis.

## Author Contributions

YD drafted the editorial and worked on the revisions with JS and DL.

### Conflict of Interest

The authors declare that the research was conducted in the absence of any commercial or financial relationships that could be construed as a potential conflict of interest.
